# Computational and Experimental Studies of Selected Types of Biomass Combustion in a Domestic Boiler

**DOI:** 10.3390/ma15144826

**Published:** 2022-07-11

**Authors:** Agnieszka Bala-Litwiniak, Dorota Musiał

**Affiliations:** Department of Production Management, Faculty of Production Engineering and Materials Technology, Czestochowa University of Technology, Armii Krajowej 19, 42-200 Czestochowa, Poland; dorota.musial@pcz.pl

**Keywords:** biomass, waste, combustion, domestic boiler, modeling

## Abstract

The paper analyzes the suitability of four types of biomass pellets as a fuel for heating purposes. Three types of waste biomass (sunflower husks, rapeseed cake, and corn straw) and one type of biomass grown for energy purposes (willow) were selected. After appropriate preparation, the selected starting materials were subjected to the pelletization process. Selected physical and chemical properties of the studied biomass pellets were determined. All four types of the analyzed pellets met the EN-ISO-17225-2:2014 standard in terms of bulk density, dimensions, as well as nitrogen and moisture content. The highest calorific value was pellets made of sunflower husk (17.27 MJ/m^3^) and willow (16.81 MJ/m^3^), while the calorific value of pellets made of corn straw and rapeseed cake did not exceed 16.5 MJ/m^3^ and did not meet the standard. In addition, the ash content for these two types of pellets was well above the standard. A 10 kW domestic biomass boiler was employed for burning the tested pellets. The consumption of analyzed fuels during boiler operation was determined. The concentration of CO, CO_2_, and NO_x_ in exhaust gases was also examined. The obtained experimental results were compared with the numerical calculations with the use of ANSYS Chemkin-Pro using two mechanisms. The highest concentrations of CO_2_ and CO were observed during the combustion of sunflower and willow husk pellets, which probably resulted from the highest carbon content and, thus, the highest calorific value when compared to cake and straw pellets. For all analyzed pellets, the value of NO and NO_2_ concentration was similar and did not exceed 368 ppm and 18 ppm, respectively. The results closest to the experiment were obtained for calculations using the mechanism developed by Glarborg et al. The research carried out in the article shows that out of the four analyzed types of pellets, only sunflower and willow husk pellets can be burned in a domestic boiler adapted to burning wood pellets, which is a cheap alternative to wood pellets.

## 1. Introduction

As it is known, the use of biomass is one of the key solutions proposed by the European Commission in order to reduce the dependence on imported fossil fuels and, thus, improve the security of the energy supply in the long term.

The main advantage of biomass is its production in the photosynthesis process, thanks to which the CO_2_ emission balance is zero, as the amount generated during biomass combustion is equivalent to the amount taken up in the photosynthesis process [1,2]. Compared to fossil fuels, biomass is a cheap and widely available renewable fuel that can be successfully used for energy production without contributing to the increase in greenhouse gas emissions, as well as in sulfur and nitrogen oxide emissions [3,4,5]. In recent years, many attempts have been made to use biomass as a fuel. For example, Radomiak et al. [6] conducted an analysis of the combustion and co-combustion of pellets made of wood biomass with glycerin waste. The results indicate that the combustion of these kinds of pellets does not practically affect CO and CO_2_ concentrations in flue gas.

In Ref. [7], the authors analyzed the combustion process of pine and sunflower pellets in a domestic boiler and their co-combustion with oats. The results showed that the co-combustion of pellets containing oats contributes to a noticeable reduction in CO and CO_2_ concentration in the flue gas.

Serrano et al. [8], in turn, analyzed the combustion efficiency and emissions in a 50 kW boiler for two species of pine from the Mediterranean Spanish forest. The parametric study showed that increasing the rate of secondary air during biomass combustion evidently reduced the CO emissions.

In Ref. [9], the authors investigated emission results for 15 kinds of biomass briquettes manufactured from a range of feedstock including hay and switch grass. The results indicate that hay and switch grass briquettes can successfully be combusted in domestic wood stoves with a similar performance and emissions to those of other woody briquettes.

As it can be seen, increasing interest has been observed in biomass-fired boilers, especially using fuel in the form of wood pellets [6,7,8,10].

The high demand for wood biomass in many industrial sectors has led to the search for other commonly available raw materials for pellet production. Based on the origin, biomass can be divided into three groups: wood biomass, waste biomass, and biomass from energy crops. In Poland, the VAT rate for wood fuels is 23%, while for nonwood pellets—8%, and for pellets made from energy crops—7%. Therefore, the production of pellets from raw materials other than wood seems to be the best economic solution [11].

One of the priorities of the European Union in terms of rational waste management is the reuse of waste. The inability to manage waste efficiently can cause serious environmental and socio-economic problems requiring urgent and reliable solutions. In response to this problem, a number of scientific initiatives were founded with the goal of creating new usages for organic residues [12,13]. One of the solutions of organic waste management can be their use for heating purposes. Agricultural waste or waste from the production of vegetable oils can be a cost-effective alternative to raw materials used for heating purposes [7,14,15]. Such waste is characterized by high calorific value, as well as zero CO_2_ emission during their combustion.

Thus, energy recovery from selected waste seems to be one of the best solutions, both in economic and environmental terms [11,16,17,18,19]. Because of the above-mentioned lower VAT on fuels produced from energy crops, it is worth considering their use as a raw material for the production of pellets. Among energy crops in Poland, willow (*Salix viminalis*) has the greatest potential, as it does not have high soil requirements, does not require large amounts of work, and is characterized by high productivity, as well as low energy consumption [20,21].

Among the raw materials from which it is possible to produce fuel pellets, one can mention production waste from the oil industry. The production of vegetable oils for both food and energy purposes generates a lot of waste with a potentially high calorific value. Especially, the management of biodiesel waste can be extremely important both economically and ecologically [22,23]. Global biodiesel production in 2019 was around 44.8 million tons, which represents 19% of the global oils and fats consumption [24]. The plant source of biodiesel usually depends on the crops amenable to the regional climate. Sunflower is one of the leading oil seed crops, cultivated for the production of oil in the world, whereas rapeseed oil is the most common source in the EU, where the share in the total area of oilseed crops is somewhat more than 80% [11,25,26]. The process of biodiesel production is predominantly carried out by catalyzed transesterification [27]. In addition to desired methyl esters, this reaction also provides a few other products, including crude glycerol and rapeseed cake or sunflower husk [28,29]. A couple of studies have demonstrated the possibility of combustion or co-combustion of crude glycerol [11,30,31,32,33,34,35]. Rapeseed cake and sunflower husk can be used as animal feed, but it often requires proper preparation of the raw material (purification and grinding), which generates additional costs. Due to the high calorific value of this waste, it can be used for heating purposes.

In recent years, the use of biomass boilers adapted for pellet burning has become an increasingly popular and environmentally friendly method of heating [30,36,37,38,39,40]. Biomass boilers are becoming increasingly more modern, allow for acceptable combustion efficiency, and are automated. The main source of biomass for the production of pellets is wood from products obtained from the wood industry. Due to the above-mentioned VAT, it is worth trying to replace wood raw materials with agriculture and oil waste, and energy crops that are broadly available and relatively cheap.

The mentioned raw materials can be an alternative to wood during pellet production; however, the chemical composition and physicochemical properties may differ from the norm, and thus have an adverse effect on the composition of exhaust gases and on the operation and maintenance of the boiler [38,41,42].

In this paper, four types of pellets made of waste and energy plants were analyzed. Three types of waste biomass (sunflower husks, rapeseed cake, and corn straw) and one type of biomass grown for energy purposes (willow) were selected. After appropriate preparation, the selected starting materials were subjected to the pelletization process. Selected physical and chemical properties of the studied biomass pellets were determined. The produced fuels were burned in a domestic heating boiler adapted to burning pellets. The concentration of CO_2_, CO, and NO_x_ was determined using an exhaust gas analyzer. The results were compared with the numerical calculation using professional software—ANSYS Chemkin-Pro. Numerical modeling of the combustion process, and, consequently, the prediction of its products, has been successfully used in many previous works [6,7,16]. The mechanism of computer simulations of gaseous pollutant formation during corn straw, rapeseed cake, sunflower husk, and willow pellets combustion is presented in the further part of the article (Section 2.3).

## 2. Research Methodology

### 2.1. Materials and Methods

One type of biomass grown for energy purposes—willow *Salix viminalis*, two types of biomass constituting waste from the production of vegetable oils—sunflower husk and rapeseed cake, and one type of agricultural waste biomass—corn straw, were selected. Biomass materials were obtained from the Trzebinia Refinery, Poland and from the L.K. farm, Radomsko, Poland.

**Figure 1:**
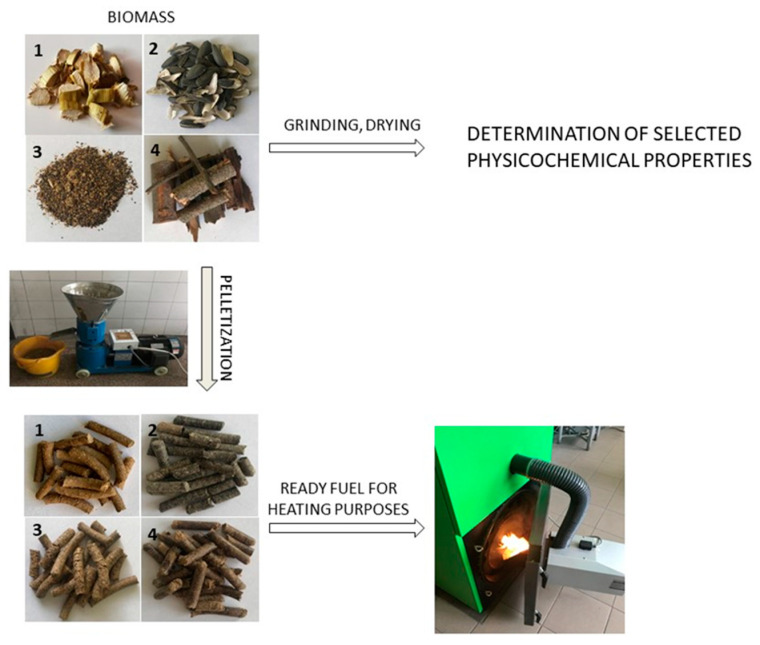
Scheme of the procedure of preparation and use of selected types of biomass as a heating fuel: 1—corn straw, 2—sunflower husk, 3—rapeseed cake, and 4—willow (*Salix viminalis*).

The analyzed raw materials, after appropriate preparation (cleaning, drying, and grounding), were subjected to the pelletization process using a KL.ZLSP pellet mill. The length and diameter of the pellets obtained were measured; the bulk density was also determined. The methodology of using selected types of biomass as a heating fuel is shown in Figure 1.

### 2.2. Test Stand Description

A 10 kW domestic biomass boiler, Mini Bio type, Kostrzewa, Poland was employed for pellet burning. The boiler was equipped with a burner adapted for the combustion of oat and biomass pellets having a diameter from 6 to 8 mm. The chamber’s internal diameter was 0.35 m and its total length was 0.5 m. The boiler water reservoir was installed around the combustion chamber and was equipped with a thermostat to maintain the water within a given temperature range. The temperature set point of the thermostat during pellet combustion was fixed at 60 °C.

The temperature inside the combustion chamber was measured using a NiCr-Ni, type K thermocouple with a temperature range from −200 °C to +1200 °C (±0.0075 × [t]). Depending on the distance from the burner, the temperature was in the range of 300–800 °C. The temperature within the representative area of the most effective burning varied between 600 °C and 700 °C. During the combustion of each of the four produced pellets, the value of the excess air coefficient was close to 2 (±0.5).

**Figure 2:**
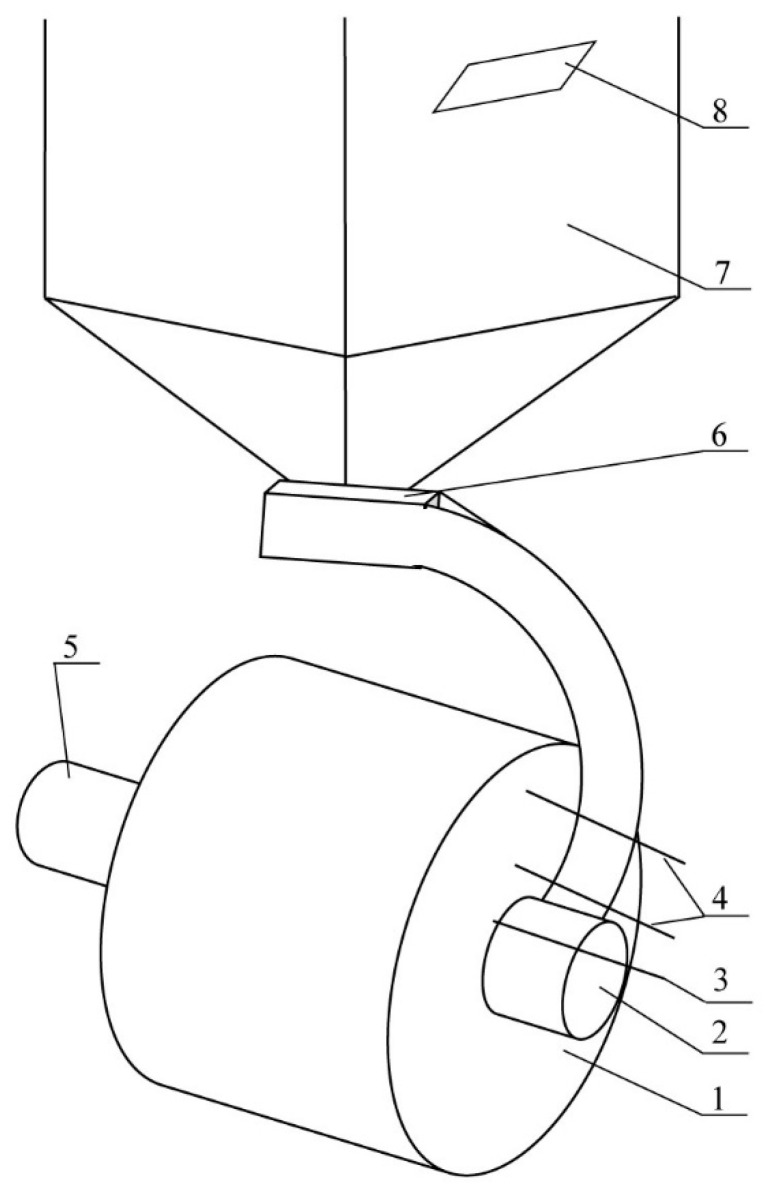
A simplified scheme of a test stand for combustion of biomass pellets: 1—combustion chamber, 2—burner Platinum Bio, 3—flue gas analyzer, 4—thermocouples, 5—gas outlet, 6—fuel feeder, 7—fuel tank, and 8—boiler operation controller.

Continuous chemical analysis of dry exhaust gases (CO_2_, CO, and NO_x_) was carried out using the Vario-Plus analyzer (MRU, Poznań, Poland) utilizing infrared sensors. The chamber’s temperature was continuously registered at the point of exhaust gas sampling by the gas analyzer. The concentrations of exhaust gases were read every 2 min, on average. The burning experiments were repeated for all four analyzed fuels 3 times and special care was taken to maintain constant boiler operating conditions, including ambient temperature and thermostat temperature setting The obtained experimental research results were compared with the numerical calculation using ANSYS Chemkin-Pro (Ansys Research 2021R1 2021.03 v3). The simplified scheme of the test stand is presented in Figure 2.

### 2.3. Modeling Procedure

Numerical simulations were performed with the application of the ANSYS Chemkin-Pro software. This software allows the solving of complex problems concerning chemical kinetics of various processes. One of the greatest advantages of the software is that the user can select the chemical mechanism used for the calculations. The use of numerical methods to predict the chemical composition of combustion products encounters several limitations resulting, among others, from:

- Significant differences between the reaction rate constants (determined on the basis of activation energies of individual combustion reactions, by different authors and for various process conditions);
- Turbulent nature of fuel combustion processes;
- Inadequacies of the existing computational models, especially visible in the simplified mechanisms assumed in CFD simulations.

Due to the large number of chemical mechanisms cited in the literature and the difficulty of specifying and selecting the proper mechanism, two mechanisms were used in the preliminary simulations:

- The one developed by Glarborg et al. [43,44] contained 107 compounds, 652 chemical reactions, and thermo-chemical and transport data;
- The one developed by The CRECK Modeling Group [45,46] contained 621 compounds, 27,829 chemical reactions, and thermo-chemical and transport data.

The obtained results from numerical simulations were reduced by the moisture content, and the shares of individual exhaust components were related to the dry exhaust gas content; thus, they could be compared with the experimental results (dry exhaust gas concentration from the analyzer).

**Figure 3:**
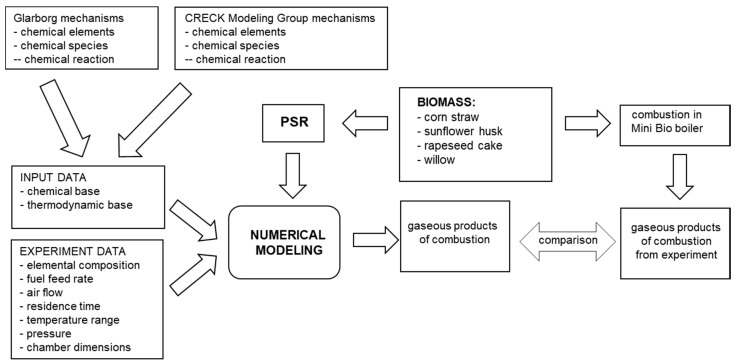
The scheme of the calculation procedure.

For preliminary measurements carried out for willow pellets, simulations were performed. The scheme of the calculation procedure is shown in Figure 3.

As shown in Figure 3, the input files used for the simulations contained kinetic data with the base of elements and compounds, as well as sets of reactions enabling the calculation of the reaction rate constants from the Arrhenius equation:

(1) $$k=ATb\cdot \exp\left(-\frac{E}{RT}\right)$$

where:

*A*—pre-exponential factor;
*b*—temperature exponent;
*E*—activation energy, J/mol;
*R*—universal gas constant = 8314 J/(mol K);
*T*—absolute temperature in the reaction zone, K.

The following boundary conditions were adopted for the calculations: fuel and air streams (Table 1) as well as elemental biomass analysis (Table 2).

A Perfectly Stirred Reactor (PSR), commonly referred to as a continuously stirred tank reactor, was used for the calculation. The selection of the chemical reactor was based on the experience of other authors who modeled the combustion and co-combustion processes of biomass [7,47,48].

**Figure 4:**
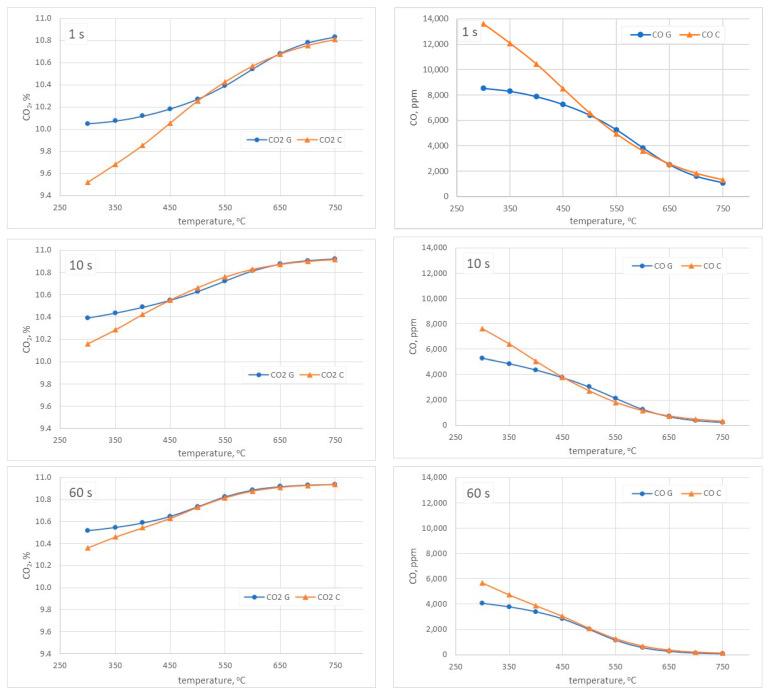
The dependence of CO_2_ and CO shares as a function of temperature for the residence time of 1–60 s.

For the PSR, the temperature range of 300–750 °C and the residence time of 1–60 s were assumed. The simulation results are shown in Figure 4. The naming of the individual mechanisms was abbreviated as follows: G—the mechanism developed by Glarborg et al.; C—the mechanism developed by The CRECK Modelling Group.

As shown in the analysis presented in Figure 4, for short residence times of the compounds in the combustion chamber (1–10 s), there are significant discrepancies between the discussed mechanisms, both in the case of CO_2_ and CO shares. For a residence time of 60 s, both mechanisms begin to show greater convergence, especially at temperatures higher than 450 °C. For CO_2_, at the temperature of 300 °C, there are differences of 0.15% between the discussed mechanisms, while at the temperature of 450 °C—at the level of 0.02%. Similarly, in the case of CO, a difference of 0.16% is observed for the lowest of the considered temperatures. At 450 °C, the differences of CO are at the level of 194 ppm, while at the highest of the analyzed temperatures, the mechanisms show greater convergence and differences at the level of 35 ppm.

The residence time of the flue gas in the combustion chamber is usually calculated as the ratio of the combustion chamber volume and the flue gas flow rate. The operating volume of the combustion chamber was not precisely known; therefore, the residence time was assumed to be 60 s as the most adequate to the real burning conditions. This is based on our earlier experience and the comparison of the preliminary numerical analysis (Figure 4) with the experimental results.

On the basis of the conducted preliminary analyses, it was determined that both mechanisms met the expectations of the conducted research.

### 2.4. Proximate and Ultimate Analyses

In order to determine the selected physicochemical properties (moisture content, ash content, calorific value, and CHN analysis), samples of the analyzed biofuels were ground in a knife mill using a sieve matrix with a mesh size of up to 1 mm.

The ash content was determined by burning a 1 g sample of all the studied biomass fuels in a muffle furnace at 250 ± 10 °C for 50 min and then at 550 ± 10 °C for 4 h, according to standard EN ISO 18122:2015. The moisture content was determined based on a 1.00 g sample weight loss after drying at 105 ± 2 °C to a constant weight according to standard EN ISO 18134-3:2015. The calorific values (combustion enthalpies) of the analyzed fuels were determined using a KL-12Mn calorimeter, PRECYZJA-BIT (Bydgoszcz, Poland), according to standard EN 14918:2009.

For each kind of tested pellet, the contents of carbon [C], hydrogen [H], and nitrogen [N] were determined using an Elemental Analyzer Truspec CHN628 LECO (St. Joseph, MI, USA). The contents of the analyzed elements were indicated by means of an infrared absorption detector (C and H) and a thermal conductivity detector (N).

## 3. Results and Discussion

As shown in Table 2, pellets made of sunflower husk and willow have the highest calorific value, while the calorific value of pellets made of corn straw and rapeseed cake do not exceed 16.5 MJ/m^3^, and, thus, they do not meet the EN ISO-17225-2:2014 standard in this respect. In addition, the ash content for these two types of pellets is well above the mentioned standard. In addition, it can be noticed that the analyzed pellets made of corn straw, sunflower husk, rapeseed cake, and willow do not exceed the standard in terms of dimensions, bulk density, and moisture content.

The requirements concerning the content of carbon and hydrogen in pellets are not specified in the standard. However, there are requirements for nitrogen, chlorine, and sulfur content. According to the standard, the nitrogen content should not exceed 1%. Therefore, the four discussed types of pellets meet the standard in this respect.

Based on a literature search [19,20,21], it was determined that the content of chlorine and sulfur in the analyzed raw materials from which the pellets were made does not exceed 0.01%, and, thus, it was assumed that the analyzed pellets were also consistent with the EN-ISO-17225-2:2014 standard in terms of the content of these elements.

### 3.1. The Results of the Numerical Calculations

**Figure 5:**
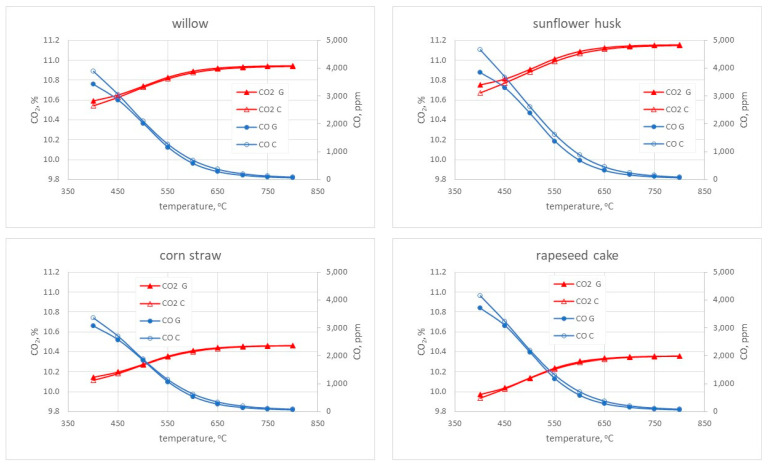
The dependence of CO_2_ and CO shares as a function of temperature for the residence time of 60 s.

**Figure 6:**
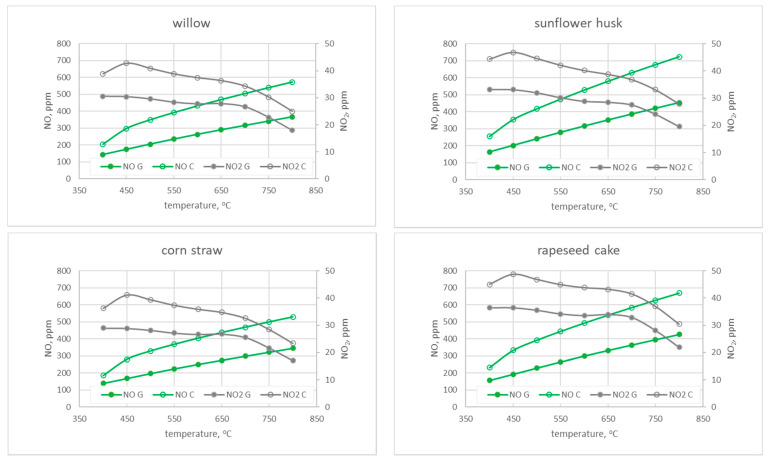
The dependence of NO and NO_2_ shares as a function of temperature for the residence time of 60 s.

Numerical calculations were carried out with the application of the ANSYS Chemkin-Pro software based on the experimental conditions fuel consumption, air stream, and elemental composition of pellets. The simulations were carried out for the temperature range observed during combustion of the analyzed pellets of 400–800 °C. For the calculations, the residence time of the reactants in the combustion chamber was assumed to be 60 s as a reference value from the first stage of calculations (Section 2.3). The results of calculating the shares of CO and CO_2_ are presented in Figure 5, while the shares of NO and NO_2_ are shown in Figure 6.

As can be seen in the presented simulation, both mechanisms implemented in the calculations show a high convergence for all types of the tested biomass, both in the case of CO_2_ and CO shares (Figure 5). Minor discrepancies can be noticed for the lowest considered temperatures. It should be noted that, for sunflower husk at 400 °C, differences of 0.082% can be seen between both mechanisms in the case of CO_2_. In the case of willow and cake, this value drops to 0.04%, and in the case of straw, it reaches a value of 0.02%. As the temperature rises, increasingly smaller discrepancies between the mechanisms are observed. The highest CO_2_ content in flue gas is observed for the sunflower husk pellet at the level of 11.2%, while the lowest is for the cake pellet at the level of 10.4%.

In addition, for the sunflower husk pellet at the temperature of 400 °C, the greatest discrepancies in the calculated CO shares are observed at the level of 800 ppm. It should be noted that the higher values were calculated using the Creck mechanism, and they reach the value of 4660 ppm. In the case of willow and cake pellets, the differences are at the level of 400 ppm, while for straw pellets, they are 300 ppm. At this temperature, the lowest amount of CO in the flue gas is observed for straw pellets at the level of 3000 ppm. It was calculated using the Glarborg mechanism. As in the case of CO_2_, the discrepancy in the share of CO between the mechanisms decreases significantly as the temperature increases. Therefore, it should be assumed that below 500 °C, proper combustion does not take place and, hence, discrepancies between the two mechanisms are visible. At the highest of the considered temperatures, differences between the mechanisms at the level of 20–30 ppm are observed for all types of biomass. In addition, for the sunflower husk pellet, the highest value of 91 ppm, calculated with the Creck mechanism, is observed.

As shown in Figure 6, the concentration of NO for all considered fuels and chemical mechanisms increases with increasing temperature. Significant differences are observed between the results obtained with both mechanisms. The highest discrepancy reaches 269 ppm at the temperature of 800 °C for sunflower husk pellets. For the other three types of biomass, these values are slightly lower at 200 ppm. At the lowest of the considered temperatures, lower discrepancies between the mechanisms, at the level of 50–90 ppm, are observed. Once again, the highest value appears in the case of sunflower husk pellets. The highest level of NO is observed at the temperature of 800 °C for sunflower husk (723 ppm for the Creck mechanism and 454 ppm for the Glarborg mechanism), and the lowest for straw (529 ppm for the Creck mechanism and 345 ppm for the Glarborg mechanism). The above dependence constitutes the reason for the different nitrogen content in the examined biomass (Table 2), whereas the amount of NO_2_ in flue gas, regardless of the type of tested biomass and the used mechanism, decreases with increasing temperature. The highest observed value at the level of 46–49 ppm is recorded for cake and sunflower husk pellets.

### 3.2. The Results of the Experiment

**Figure 7:**
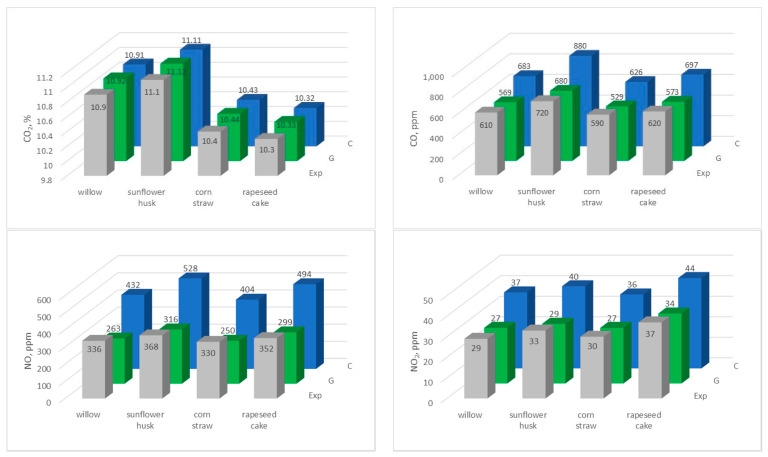
A comparative analysis of the experimental and computational results for the temperature of 600 °C and the residence time of 60 s.

The obtained results of the experiment were compared with the results of the calculations carried out for a residence time of 60 s and a temperature of 600 °C (Figure 7).

The comparative analysis of experimental data and results of numerical calculations shows a high conformity. Especially for CO_2_ values, it is possible to observe an almost 100% conformity of the results, both those measured with a flue gas analyzer (Exp), as well as those obtained as a result of calculations using the mechanisms developed by Glarborg (G) and the CRECK Modelling Group (C). When analyzing the concentration of CO, NO, and NO_2_, we can observe, above all, significant calculation differences between these two mechanisms. As a greater convergence between the experimental results and the calculations is observed for the G mechanism, it can be concluded that this mechanism is closer to the conditions of the experiment. By analyzing the number of reactions adopted in individual mechanisms, it can be assumed that the G mechanism, which contains over 40 times fewer reactions compared to the C mechanism, reflects reality better. Therefore, focus is put on the comparative analysis between the results of the experiment and the calculations resulting from the G mechanism. Therefore, there is a high convergence for the concentrations of CO and NO, but the concentrations measured by the flue gas analyzer are slightly higher than those resulting from the calculations taking the G mechanism into account. However, an inverse dependency can be observed for the concentration of NO_2_, of which the experiment results in 2–3-fold lower values. These differences probably result from the assumption of a perfect mixing of the reagents used in the calculations, while the concentration from the experiment constitutes an instantaneous value measured at one point (number 6 in Figure 2). It can be assumed that, if the experimental data were collected from several measurement points and averaged, it would be close to the values obtained during calculations. The higher values of CO concentrations measured during combustion in the boiler should be explained in the same way. The PSR reactor used for the calculations assumes a perfect mixing of reactants, thanks to which CO is completely burnt, which cannot be said about the experiment. Furthermore, it should be noted that the chemical mechanism used to calculate nitrogen compound formation was complex (including N_2_O), while only NO and NO_x_ concentrations were measured during the experiment. In the comparative analysis, it was assumed that the NO_2_ share constitutes the difference between NO_x_ and NO. When analyzing the individual types of pellets in terms of exhaust emissions, the highest concentrations of CO_2_ and CO are observed during the combustion of willow and sunflower husk pellets, which is probably due to their highest carbon content and, thus, higher calorific value compared to rapeseed cake and straw pellets. For all analyzed pellets, the value of NO and NO_2_ concentration is similar, and does not exceed 368 ppm and 18 ppm, respectively.

## 4. Conclusions

In the times of rising prices and limited availability of fossil fuels, it is of great im-portance to search for new, cheaper solutions, especially in the area of fuels obtained from widely available biomass, including waste biomass. However, not all fuels of this type meet the standards in terms of the appropriate calorific value or ash content. Finding alternatives to fossil fuels is extremely important both economically and ecologically.

The following conclusions were drawn from the analyses carried out in the article:

- All four types of the analyzed pellets meet the EN-ISO-17225-2:2014 standard in terms of bulk density, dimensions, as well as nitrogen and moisture content. The ash content for corn straw (7.56%) and rapeseed cake (5.77%) pellets is well above the aforementioned standard, according to which the ash content for this type of fuel should not exceed 3%.
- The highest concentrations of CO_2_ and CO are observed during the combustion of sunflower and willow husk pellets, which probably results from the highest carbon content and, thus, the highest calorific value when compared to cake and straw pellets.
- Too high an ash content and, at the same time, low calorific value do not make corn straw and rapeseed cake pellets good fuels, especially one that could be burned in biomass-fired domestic heating boilers.
- The research carried out in this article shows that, out of the four analyzed types of pellets, only sunflower husk and willow pellets can be combusted in this type of boiler and, at the same time, constitute a cheap alternative to wood pellets.
- The two mechanisms used in the numerical calculations show high convergence for CO_2_ concentration (±0.01%) and significant discrepancies in terms of NO and CO concentrations (average of 130 ppm for NO and 180 ppm for CO). The results most similar to the experiment were obtained for the calculations using the mechanism developed by Glarborg et al. The dependence between the discrepancies concerning the results of the two considered mechanisms requires further, detailed analysis.

## Figures and Tables

**Table 1:** Comparison of fuel feed rate and air flow during combustion of straw, sunflower husk, rapeseed cake, and willow pellets.

Fuel	Fuel Feed Rate, kg/s	Air Flow, m^3^/s
Corn straw	0.000500	0.003850
Sunflower husk	0.000306	0.002316
Rapeseed cake	0.000417	0.00327
Willow	0.000333	0.002522

**Table 2:** A comparison of the selected physicochemical properties and elemental composition for pellets made of straw, sunflower husk, rapeseed cake, and willow.

Analyzed Parameters	Corn Straw	Sunflower Husk	Rapeseed Cake	Willow	EN ISO-17225-2:2014
Proximate analysis					
Moisture, % (±0.1%)	5.31	4.55	9.29	5.82	≤10
Ash, % (±0.1%)	7.56	2.45	5,77	1.96	≤3
Ultimate analysis					
C, % (±0.1%)	46.7	48.7	47.5	48.1	-
H, % (±0.1%)	6.3	5.9	6.0	5.9	-
N, % (±0.1%)	0,6	1.0	0.9	0.7	≤1
O, % (bal.)	33.53	36.4	30.54	37.52	-
Calorific value, MJ/kg (±0.1 MJ/kg)	15.57	17.27	16.23	16.81	≥16.5
Bulk density, kg/m^3^ (±10 kg/m^3^)	500	585	575	550	≥500
Length, mm (±1 mm)	5–15	5–15	5–15	5–15	3.15–40
Diameter, mm (±1 mm)	6	6	6	6	6

## Data Availability

The data presented in this study are available on request from the corresponding author.
